# Monocyte distribution width compared with C-reactive protein and procalcitonin for early sepsis detection in the emergency department

**DOI:** 10.1371/journal.pone.0250101

**Published:** 2021-04-15

**Authors:** A la Woo, Dong Kyu Oh, Chan-Jeoung Park, Sang-Bum Hong

**Affiliations:** 1 Department of Pulmonary and Critical Care Medicine, Asan Medical Center, University of Ulsan College of Medicine, Seoul, Republic of Korea; 2 Department of Laboratory Medicine, Asan Medical Center, University of Ulsan College of Medicine, Seoul, Republic of Korea; Heidelberg University Hospital, GERMANY

## Abstract

**Purpose:**

Monocyte distribution width (MDW) has been suggested as an early biomarker of sepsis, but few studies have compared MDW with conventional biomarkers, including C-reactive protein (CRP) and procalcitonin (PCT). This study evaluated MDW as a biomarker for sepsis and compared it with CRP and PCT.

**Materials and methods:**

Patients aged 18–80 years who visited the emergency department were screened and prospectively enrolled in a tertiary medical center. Complete blood count, MDW, CRP, and PCT were examined. Diagnostic performance for sepsis was tested using the area under the curve (AUC) of receiver operating characteristic (ROC) curves, sensitivity, and specificity.

**Results:**

In total, 665 patients were screened, and 549 patients with valid laboratory test results were included in the analysis. The patients were categorized into three groups according to the Sepsis-3 criteria: non-infection, infection, and sepsis. MDW showed the highest value in the sepsis group (median [interquartile range], 24.0 [20.8–27.8]). The AUC values for MDW, CRP, PCT, and white blood cells for predicting sepsis were 0.71 (95% confidence interval [CI], 0.67–0.75), 0.75 (95% CI, 0.71–0.78], 0.76 (95% CI, 0.72–0.79, and 0.61 (95% CI, 0.57–0.65), respectively. With the optimal cutoff value of the cohort, the sensitivity was 83.0% for MDW (cutoff, 19.8), 69.7% for CRP (cutoff, 4.0), and 76.6% for PCT (cutoff, 0.05). The combination of quick Sequential Organ Failure Assessment (qSOFA) with MDW improved the AUC (0.76; 95% CI, 0.72–0.80) to a greater extent than qSOFA alone (0.67; 95% CI, 0.62–0.72).

**Conclusions:**

MDW reflected a diagnostic performance comparable to that of conventional diagnostic markers, implying that MDW is an alternative biomarker. The combination of MDW and qSOFA improves the diagnostic performance for early sepsis.

## Introduction

Sepsis is a common reason for patients to visit the emergency department (ED), hospital admission, and intensive care unit (ICU) mortality [1]. As the early administration of antibiotics results in better clinical outcomes [2–4], earlier detection helps in developing better sepsis management strategies.

As the diagnosis of sepsis is made by clinical definition, several pieces of clinical information are required for sepsis diagnosis. The detection of sepsis is often delayed because of the complexity of the Sequential Organ Failure Assessment (SOFA). To overcome the complexity of the SOFA, the Sepsis-3 group [5] has suggested using the quick SOFA (qSOFA) for rapid assessment. Although the qSOFA is a good indicator of mortality, it has limitations as a screening tool in the ED [6]. Therefore, using biomarkers in sepsis detection may be beneficial in decision making for further evaluation of sepsis. However, no biomarkers have been recommended in the current sepsis guidelines.

Monocytes are one of the first responders to infection in the blood and peripheral tissue and display functional and volumetric heterogeneity [7]. During sepsis, functional reprogramming between monocytes and macrophages occurs [8], inducing morphological changes in the monocytes; monocyte distribution width (MDW) reflects those changes and has been suggested as a marker of early sepsis in the ED [9, 10]. However, few studies have compared MDW with C-reactive protein (CRP) and procalcitonin (PCT), well-known biomarkers of sepsis [11].

Hence, this study evaluated the diagnostic value of MDW in predicting sepsis and compared it with those of other biomarkers, such as white blood cell (WBC) count, CRP, and PCT, and clinical scores (qSOFA) for detecting early sepsis.

## Materials and methods

### Study design and population

From November 1, 2018 to July 1, 2019, patients who visited the ED of a tertiary teaching hospital (Asan Medical Center, Seoul, Korea) were prospectively enrolled. Those aged 18–80 years were enrolled if the physician ordered a complete blood count (CBC) with differential tests for any reason. Patients who were pregnant, were previously enrolled in this study due to multiple visits, and had invalid laboratory results (CBC with differential tests without-of-range events, invalid pulses, or partial clogs; CBC with differential tests and MDW with the presence of “R” flag for differential results; non-reportable MDW because of excessive debris, low monocyte count [less than 100/ μL]) were excluded. The patients were followed up during ED stay, and the SOFA scores of the patients were assessed two times during ED stay (initial and final SOFA scores), if available (in some cases, only one value was available because of the short ED stay). We used the worst SOFA score during ED stay to define sepsis. The systemic inflammatory response syndrome (SIRS) score was assessed as well. Then, the patients were categorized into six groups: control (non-infection), infection, SIRS, sepsis, severe sepsis, and septic shock according to the Sepsis-2 criteria [12]. The description of each group is as follows: (1) non-infection, patients without any evidence of infection and zero or one component of the SIRS criteria; (2) infection, patients with evidence of infection but zero or one component of the SIRS criteria, (3) SIRS; (4) sepsis, patients with infection and SIRS; (5) severe sepsis, patients with sepsis and evidence of organ dysfunction or tissue hypoperfusion; and (6) septic shock, patients with severe sepsis, and hypotension not reversed with fluid resuscitation. After patient enrollment and data collection, we recategorized the patients enrolled into three groups: non-infection, infection, and sepsis according to the Sepsis-3 criteria [5]. The description of each group is as follows: (1) non-infection, patients without any evidence of infection; (2) infection, patients with evidence of infection (positive blood culture or serology test also when the clinician suspected infection by clinical diagnosis); and (3) sepsis, patients with evidence of infection with the SOFA score being higher than two points, according to the Sepsis-3 criteria [5]. Thus, we could examine the effectiveness of MDW and other biomarkers using both the Sepsis-2 and Sepsis-3 criteria. The subgroup analysis for diagnostic performance was performed according to the patients’ immune statuses. Immune-compromised patients were those with malignancy, those with neutropenic status (neutropenia defined when the neutrophil count was less than 1,500/μL [13]), those diagnosed with acquired immunodeficiency syndrome, or those taking immune-suppressant agents due to solid organ or bone marrow transplantation.

### Data collection and laboratory marker measurement

We collected the clinical data (e.g., medical history, results of imaging test, results of culture studies, vital signs, management in the ED, and medication details, among others) from the electronic medical record and the results of the laboratory tests in the ED. Blood samples collected in di-potassium (K2) ethylene diamine tetraacetic acid (EDTA) tubes (at a controlled room temperature) within 4 h from blood draw [14] at the ED were used in the sample analysis. MDW was measured using an automatic blood cell analyzer DxH 900 (Beckman Coulter, Inc, Brea, California, USA) with the blood sample taken for baseline CBC test. The manufacturer’s recommended blood sample for analysis was within 2 h from venipuncture [15]. We planned to include blood samples obtained within 4 h for the stability of MDW results at room temperature. The CRP (Roche CRPL3 by Cobas c702 module, Roche Diagnostics, Basel, Switzerland) and PCT (ADVIA Centaur Procalcitonin by ADVIA Centaur XPT, Siemens Healthcare Diagnostics Inc. Pennsylvania USA) results were obtained from the blood in serum separate tubes collected within 4 h at the ED.

### Study outcome and statistical analysis

The primary aim of this study was to evaluate and compare the diagnostic accuracy of biomarkers using the area under the curve (AUC) of receiver operating characteristic (ROC) curves. The secondary aim was to examine the diagnostic performance of the combination of biomarkers and clinical score. We used Statistical Package for the Social Sciences version 21.0 (IBM Corp., Armonk, NY, USA) and MedCalc^®^ (MedCalc Software, Ostend, Belgium) in this analysis. Continuous variables were presented as mean ± standard deviation (SD) (for normally distributed variables) or median with interquartile ranges (IQR) (for non-normally distributed variables). Categorical variables were presented as percentage with numbers. To compare baseline characteristics of the three groups for the Sepsis-3 criteria and four groups for the Sepsis-2 criteria, analysis of variance was used for continuous variables with normal distribution and the chi-square test or Fisher’s exact test was used for categorical variables. To determine the optimal cutoff values of MDW, CRP, and PCT, we used Youden’s index from ROC curve analysis in a univariate model. The diagnostic performance of biomarkers was assessed in terms of sensitivity, specificity, positive predictive value, negative predictive value, positive likelihood ratio, and negative likelihood ratio with 95% confidence intervals (CIs) using cutoff values derived from a previous cohort trial [10] or the optimal cutoff values obtained from this study’s cohort. Comparison of diagnostic performance among inflammatory markers and qSOFA scores for predicting sepsis was performed using the AUC of ROC curve with 95% CIs. The results were considered statistically significant when the two-tailed *p*-value was less than 0.05.

### Ethical approval

We conducted the study according to the principles of the Declaration of Helsinki. The local regional ethics board (the Ethics Committee of the Asan Medical Center; Institutional Review Board No. 2018–1159) approved this study. Because the patients enrolled in this study were under emergency circumstances and the study was not an interventional study, the IRB waived the requirement for informed consent. All data were anonymized before access by the authors.

## Results

### Patient characteristics

**Fig 1:**
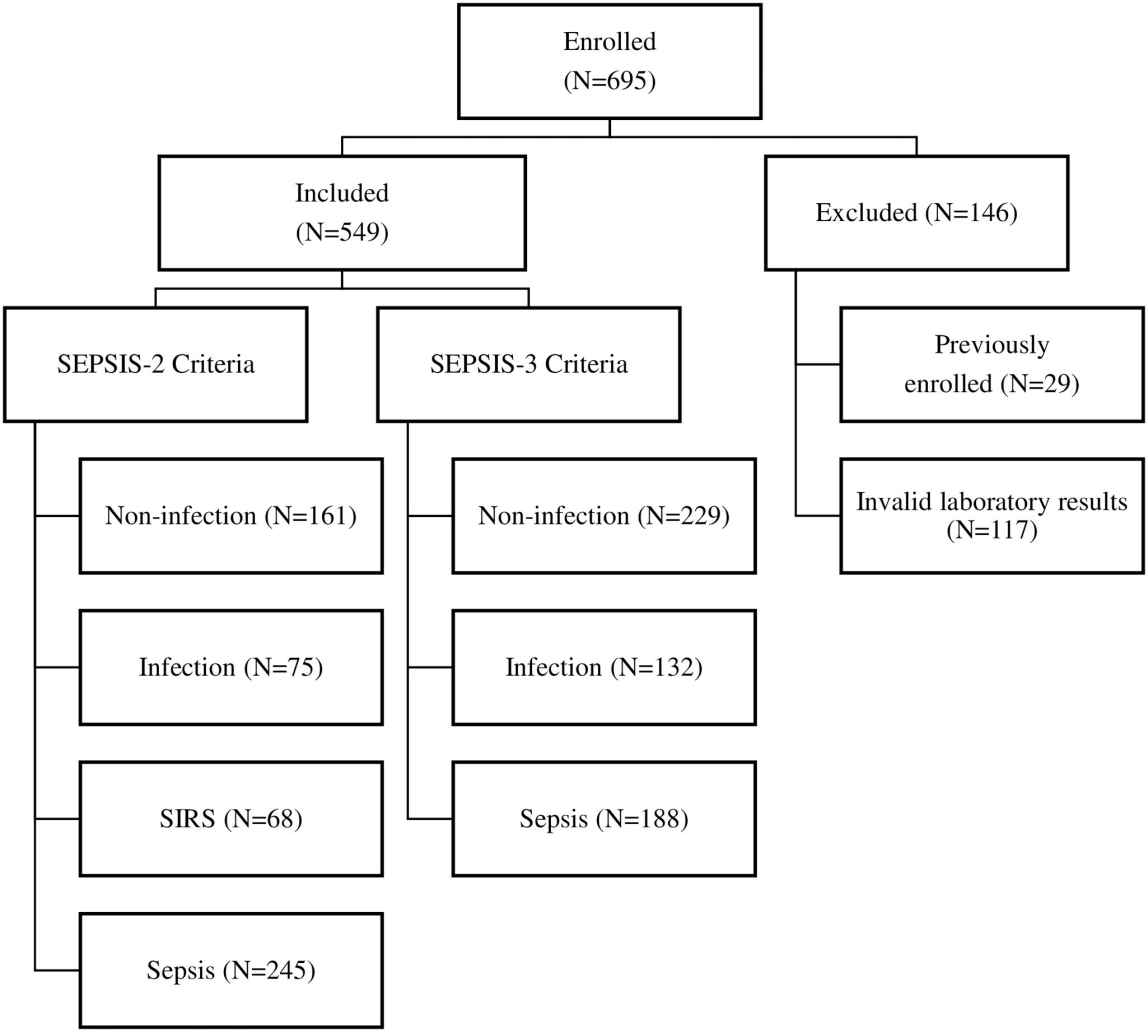
Flow of the study.

During the study period, 695 patients were enrolled when the physician recommended CBC with differential counts in the ED, among whom, patients who were pregnant or previously enrolled (due to the multiple ED visits during the study period) or those with invalid laboratory results were excluded. Finally, 549 patients with CBC, MDW, CRP, and PCT data were included in the analysis in this study (Fig 1).

**Fig 2:**
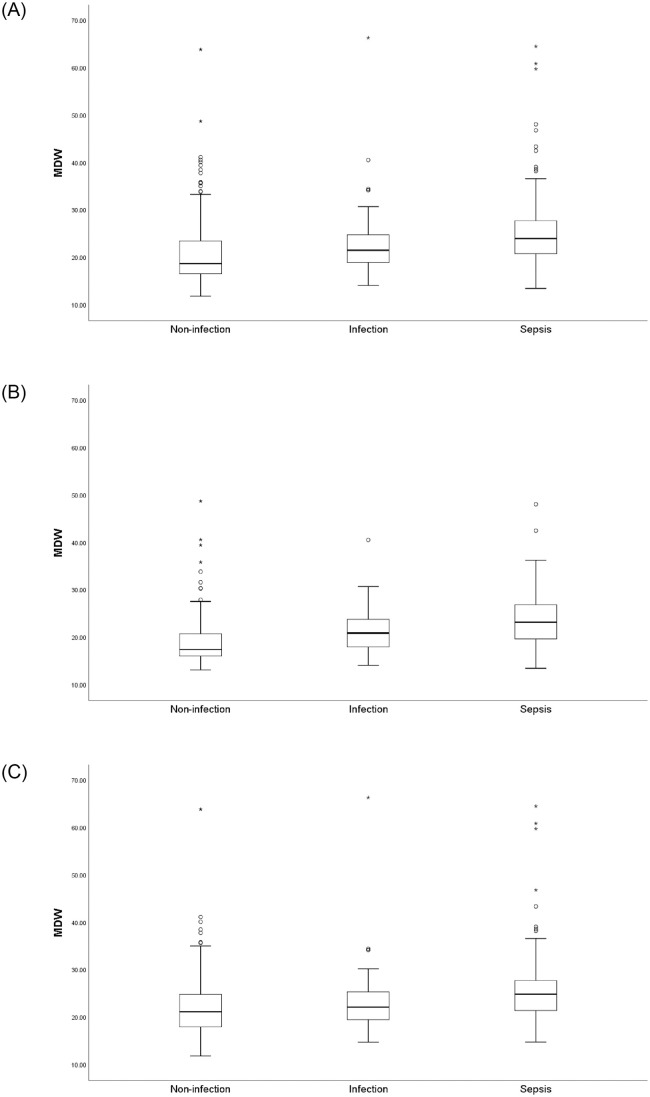
Comparison of MDW among groups with different immune statuses according to Sepsis-3 definition. (A) All patients. (B) Immune-competent patients. (C) Immune-compromised patients. MDW, monocyte distribution width.

Baseline characteristics of the overall population according to the Sepsis-3 classification are presented in Table 1. The mean age of the overall population was 59.2 years (SD, 13.3), and male patients accounted for 55% of the overall population. Among all patients, 267 (48.6%) had at least one active malignancy, of whom, 126 (23%) had metastatic disease, 19 (3.5%) had hematologic malignancy, and 117 (21.3%) were treated with cytotoxic chemotherapy within 2 weeks before ED visit. The median SOFA score of the cohort at the ED was 1 (IQR, 1–3). The sepsis group accounted for 34.2% of the overall population (*n* = 188). Of those, 9.6% (n = 18) had septic shock. All inflammatory biomarkers showed the highest value in the sepsis group (Table 2 and S2 Table). The median MDW value showed an increasing trend when the immune status was compromised (S2 Table and Fig 2).

**Table 1:** Baseline clinical characteristics of patients visiting the emergency department.

	Total (n = 549)	Non-infection (n = 229)	Infection (n = 132)	Sepsis (n = 188)	*P*-value
Age, years, mean (SD)	59.2 (13.3)	57.8 (13.8)	55.5 (13.9)	63.4 (11.0)	< 0.001
Sex, male, n (%)	302 (55.0)	118 (51.5)	74 (56.1)	110 (58.5)	0.348
Previous medical condition, n (%)					
Malignancy	267 (48.6)	79 (34.5)	71 (53.8)	117 (62.2)	< 0.005
Neutropenia^a^	67 (12.2)	20 (8.7)	19 (14.4)	28 (14.9)	0.109
Use of antibiotics^b^, n (%)	73 (13.3)	14 (6.1)	23 (17.4)	36 (19.1)	< 0.001
Use of G-CSF, n (%)	49 (8.9)	12 (5.2)	16 (12.1)	21 (11.2)	0.036
Immune-compromised, n (%)					
HIV or organ transplant	6 (1.1)	3 (1.3)	0 (0)	3 (1.6)	0.368
Chemotherapy^c^	117 (21.3)	34 (14.8)	30 (22.7)	53 (28.2)	0.004
CMI, median (IQR)	4 (2–6)	3.0 (1.0–5.0)	3.0 (2.0–6.0)	5.0 (4.0–7.8)	< 0.001
SOFA score^d^, median (IQR)	1 (1–3)	1 (1–2)	1 (1–1)	3 (2–5)	< 0.001
qSOFA^d^, median (IQR)	0 (0–1)	0 (0–0)	0 (0–1)	1 (0–1)	< 0.001
Lactic acid^e^ (mmol/L), median (IQR)	1.4 (1.0–2.0)	1.4 (1.0–1.9)	1.1 (0.8–1.4)	1.6 (1.1–2.1)	< 0.001

SOFA, Sequential Organ Failure Assessment; qSOFA, quick Sequential Organ Failure Assessment; ED, emergency department; G-CSF, granulocyte colony-stimulating factor; IQR, interquartile range; SD, standard deviation; CMI, Charlson Comorbidity Index.

^a^Neutropenia was defined as neutrophil counts of less than 1,500/μL [13].

^b^Use of antibiotics was defined as using antibiotics within 7 days before ED visit.

^c^Chemotherapy was defined as taking any cytotoxic chemotherapy within 2 weeks before emergency department visit.

^d^Scores were the values calculated during ED admission.

^e^Lactic acid was the initial value at the ED.

**Table 2:** Comparison of biomarker levels according to Sepsis-3 definition.

	Total	Non-infection (n = 229)	Infection (n = 132)	Sepsis (n = 188)	*P*-value
CRP (mg/dL), median (IQR)	3.3 (0.49–9.08)	0.52 (0.1–3.66)	4.51 (1.21–10.02)	6.70 (2.79–14.22)	< 0.001
PCT (ng/mL), median (IQR)	0.05 (0.05–0.31)	0.05 (0.05–0.098)	0.05 (0.05–0.15)	0.22 (0.06–1.05)	< 0.001
WBC (×10^3^/μL), median (IQR)	8.2 (5.6–11.6)	7.5 (5.3–10.7)	8.8 (6.3–12.4)	9.2 (5.9–14.0)	0.001
MDW, median (IQR)	21.5 (18.0–25.8)	18.7 (16.6–23.5)	21.5 (19.0–24.8)	24.0 (20.8–27.8)	< 0.001

MDW, monocyte distribution width; WBC, white blood cell; CRP, C-reactive protein; PCT, procalcitonin; IQR, interquartile range.

### Diagnostic performance according to Sepsis-3 definition

**Fig 3:**
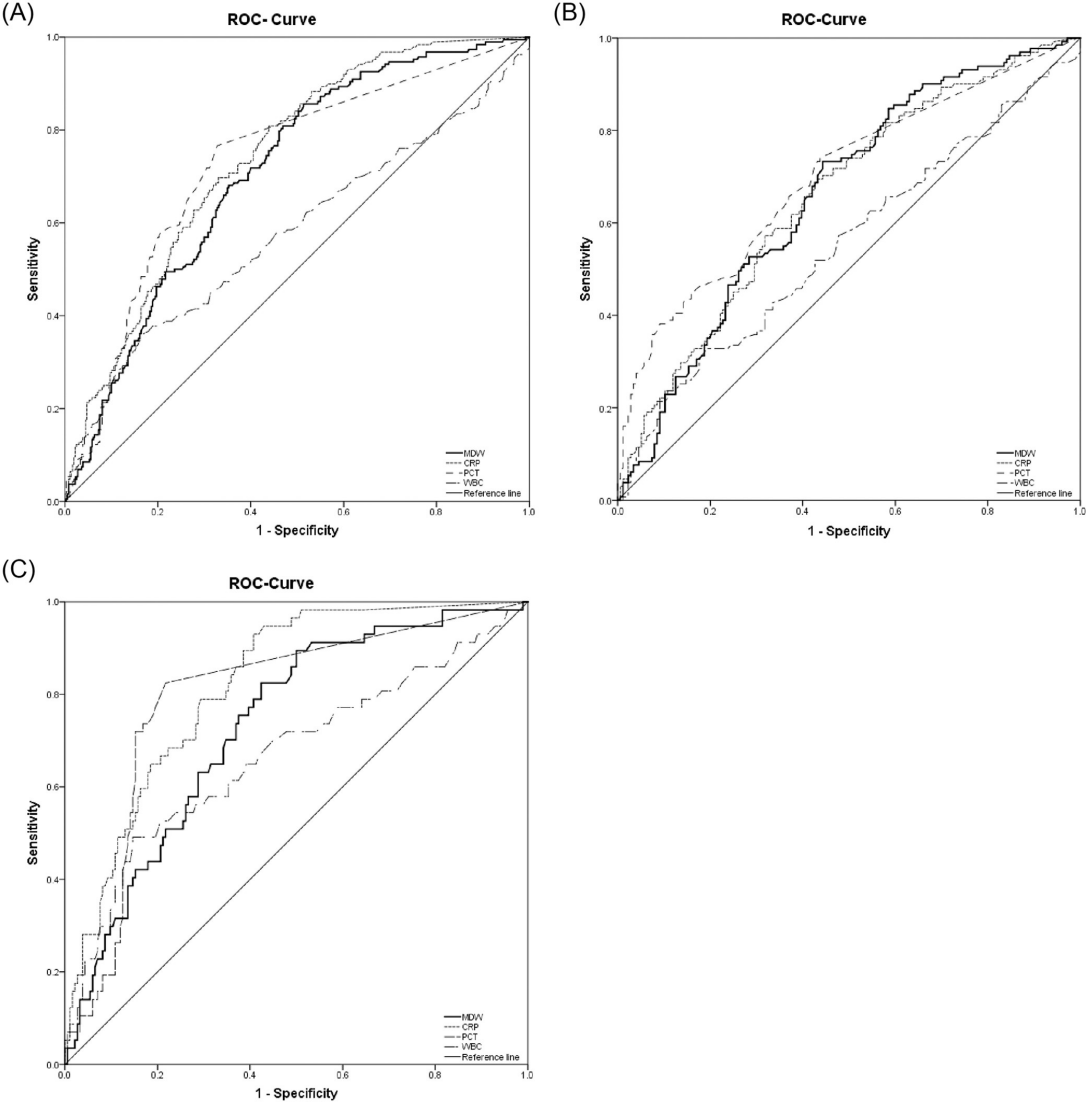
AUC of ROC curve for sepsis prediction, according to immune status. (A) Comparison of AUROC in the overall population. WBC count alone is inferior to other biomarkers. (B) Comparison of AUROC in immune-compromised patients. (C) Comparison of AUROC in immune-competent patients. AUROC is relatively higher in immune-competent patients. ROC, receiver operating curve; AUC, area under the curve; AUROC, area under the curve of receiver operating curve; MDW, monocyte distribution width; CRP, C-reactive protein; PCT, procalcitonin; WBC, white blood cell.

The discriminatory function of MDW in predicting sepsis (using sepsis-3 definition) was significantly different from that of WBC count but did not show a significant difference from those of CRP and PCT (Fig 3 and Table 3). MDW with a cutoff value of 19.8 showed the highest sensitivity of 83.0% and negative predictive value of 84.9% among the tested biomarkers (CRP, 69.7% and 80.9% with a cutoff value of 4.0 mg/dL, and PCT, 76.6% and 84.6% with a cutoff value of 0.05 ng/mL) (Table 3), which was observed when the cutoff value of 20.0, obtained from a previous study [10].

**Table 3:** Diagnostic performance of WBC count, CRP, PCT, and MDW in predicting sepsis (by Sepsis-3 definition).

	MDW		CRP	PCT	WBC
AUC (95% CI)	0.71 (0.67–0.75)		0.75 (0.71–0.78)	0.76 (0.72–0.79)	0.61* (0.57–0.65)
Cutoff Value	19.8 ^a^	20.0^b^	4.0 ^a^	0.05^a^	<4,000/μL or >12,000/μL^c^
Sensitivity, % (95% CI)	83.0 (76.8–88.1)	81.4† (75.1–86.7)	69.7 (62.6–76.2)	76.6 (69.9–82.3)	49.5 (42.1–56.8)
Specificity, % (95% CI)	49.9 (44.6–55.1)	50.7† (45.4–56.0)	67.0 (61.9–71.9)	67.2 (62.1–72.1)	72.6 (67.7–77.1)
Positive predictive value, % (95% CI)	34.2 (30.3–38.4)	46.2† (43.1–49.3)	52.4 (48.0–56.7)	55.0 (50.8–59.1)	48.4 (43.0–54.0)
Negative predictive value, % (95% CI)	84.9 (80.1–88.7)	83.9† (79.2–87.8)	80.9 (77.2–84.2)	84.6 (80.8–87.8)	73.4 (70.3–76.3)
Positive LR (95% CI)	1.65 (1.47–1.87)	1.65† (1.46–1.87)	2.11 (1.77–2.52)	2.34 (1.98–2.76)	1.80 (1.45–2.25)
Negative LR (95% CI)	0.34 (0.24–0.48)	0.37† (0.27–0.50)	0.45 (0.36–0.57)	0.35 (0.27–0.46)	0.70 (0.60–0.81)

MDW, monocyte distribution width; WBC, white blood cell; CRP, C-reactive protein; PCT, procalcitonin; CI, confidence interval; LR, likelihood ratio.

^a^The cutoff value was adopted from the value maximizing Youden’s index.

^b^Adopted from a previous study.

^c^The cutoff value for WBC count was adapted from systemic inflammatory response syndrome.

*The AUC is significantly different from that of MDW.

^†^The value derived using the cutoff value from a previous study (20.0).

When we excluded immune-compromised patients, MDW of immune-competent patients indicated an AUC of 0.73 (95% CI, 0.66–0.80). Other biomarkers demonstrated an AUC of 0.82 (95% CI, 0.76–0.88) for CRP, 0.80 (95% CI, 0.77–0.88) for PCT, and 0.67 (95% CI, 0.62–0.76) for WBC count (Table 4).

**Table 4:** Diagnostic performance of WBC count, CRP, PCT, and MDW for predicting sepsis according to immune status.

	MDW	CRP	PCT	WBC count
Immune-compromised^c^ (n = 307)				
AUC (95% CI)	0.66 (0.60–0.72)	0.66 (0.60–0.72)	0.70 (0.64–0.76)	0.56 (0.50–0.63)*
Cutoff value	22.0^a^	4.0^a^	0.05^a^	<4000/μL or >12,000/μL^b^
Sensitivity (95% CI)	73.3 (64.9–80.6)	70.2 (61.6–77.9)	74.1 (65.7–81.3)	48.9 (40.0–57.7)
Specificity (95% CI)	55.1 (47.5–62.6)	55.7 (48.0–63.1)	56.3 (48.6–63.7)	63.1 (55.5–70.2)
Immune-competent^d^ (n = 242)				
AUC (95% CI)	0.73 (0.66–0.80)	0.82 (0.76–0.88)	0.80 (0.77–0.88)	0.67 (0.62–0.76)
Cutoff value	19.0^a^	4.0^a^	0.05^a^	<4000/μL or >12,000/μL^b^
Sensitivity (95% CI)	82.5 (70.1–91.3)	67.4 (54.8–80.1)	82.5 (70.1–91.3)	50.9 (37.3–64.4)
Specificity (95% CI)	57.8 (50.4–65.1)	77.8 (71.2–83.6)	78.3 (71.6–84.0)	81.6 (75.3–86.9)

MDW, monocyte distribution width; WBC, white blood cell; CRP, C-reactive protein; PCT, procalcitonin; CI, confidence interval.

^a^The cutoff value was adopted from the value maximizing Youden’s index.

^b^The cutoff value for WBC count was adapted from the definition of systemic inflammatory response syndrome.

^c^Immune-competent was defined as patients not immune-compromised.

^d^Immune-compromised is defined as patients with any malignancy, who were treated with G-CSF, with neutropenia, who underwent organ transplantation, or with acquired immunodeficiency syndrome.

*The AUC is significantly different from that of MDW.

In immune-compromised patients, no significant difference in AUC among all biomarkers studied—0.66 (95% CI, 0.62–0.71) for MDW, 0.66 (95% CI, 0.61–0.72) for CRP, and 0.70 (95% CI, 0.64–0.76) for PCT, except for WBC count. A decreasing trend in the AUC of the biomarkers studied were observed in immune-compromised patients compared with those in immune-competent patients (Table 4).

### Combination of biomarkers and clinical score

We tested the diagnostic performance of the combination of MDW and other biomarkers in predicting sepsis. When MDW was combined with WBC count, CRP, or PCT, no improvement in the AUC of ROC curve was observed (S3 Table). However, when combined with qSOFA, MDW showed a significantly improved diagnostic performance in all patients (Table 5).

**Table 5:** Comparison of discriminative performance of biomarkers combined with qSOFA.

	qSOFA	MDW and qSOFA	WBC count and qSOFA	CRP and qSOFA	PCT and qSOFA	qSOFA, WBC count, and MDW
All patients (n = 549)						
AUC (95% CI)	0.67 (0.62–0.72) †	0.76 (0.72–0.80)*	0.69 (0.64–7.34)	0.77 (0.73–0.81)*	0.76 (0.72–0.81)*	0.78 (0.74–0.82)*
Immune-competent^a^ (n = 242)						
AUC (95% CI)	0.70 (0.62–0.78) †	0.70 (0.61–0.79)	0.68 (0.58–0.77)	0.74 (0.65–0.82)	0.70 (0.61–0.79)	0.71 (0.62–0.80)
Immune-compromised^b^ (n = 307)						
AUC (95% CI)	0.66 (0.59–0.72) †	0.74 (0.69–0.80)	0.66 (0.60–0.73)	0.71 (0.66–0.77)	0.74 (0.69–0.80)	0.74 (0.69–0.80)

MDW, monocyte distribution width; WBC, white blood cell; CRP, C-reactive protein; PCT, procalcitonin; CI, confidence interval; qSOFA, quick Sequential Organ Failure Assessment.

^a^Immune-competent was defined as patients not immune-compromised.

^b^Immune-compromised is defined as patients with any malignancy, who were treated with G-CSF, with neutropenia, who underwent organ transplantation, or with acquired immunodeficiency syndrome.

*The AUC is significantly different from that of qSOFA.

^†^The AUC of SOFA is not significantly different from that of MDW (Tables 3 and 4).

### Diagnostic performance according to sespsis-2 definition

Baseline characteristics of patients according to the Sepsis-2 criteria and diagnostic performance of the biomarkers studied in predicting sepsis are presented in the supporting information file (S4 and S5 Tables, respectively). Based on Sepsis-2 definition, 245 patients were categorized as sepsis (*n* = 245, 44.6%). The diagnostic performance was demonstrated as AUC (95% CI) of 0.70 (0.66–0.74) for MDW, 0.69 (0.65–0.72) for WBC count, 0.72 (0.68–0.76) for PCT, and 0.79 (0.75–0.82) for CRP.

## Discussion

In this study, we tested the diagnostic performance of MDW in predicting sepsis and compared it with those of conventional biomarkers (CRP, PCT, and WBC count). The diagnostic performance of MDW was presented in AUC (0.71; 95% CI, 0.67–0.75) and compared with those of CRP (0.75; 95% CI, 0.71–0.78), PCT (0.76; 95% CI, 0.72–0.79), and WBC count (0.61; 95% CI, 0.57–0.65). In this study, the cutoff value of MDW was 19.8, which is similar to 20.0 [10] and 20.5 [9] observed in other studies. Furthermore, MDW showed an 83.0% sensitivity with its optimal cutoff value from the study cohort, which was higher than those of CRP (69.7%) and PCT (76.6%).

Despite the SOFA score being good for differentiating the prognosis of sepsis, the time for gathering the information needed to assess the SOFA may impede early sepsis diagnosis. In this regard, many endeavors have been faced in finding a simple screening tool that would act as a good biomarker of sepsis [16–19], but none of them had shown superiority over others. Among the numerous biomarkers recommended for predicting sepsis, CRP and PCT are the most widely used in clinical practice [19]. CRP is an acute-phase reactant produced by hepatocytes and increases within the first 6–8 h and peaks after 48 h. It has been suggested as a sepsis marker [20] but has been found to increase in various medical conditions [21]. PCT is a good biomarker of sepsis [22] and increases earlier than CRP [23]; it also has an advantage in managing antibiotics [24].

In this study, we analyzed the efficacy of MDW in ED based on the Sepsis-3 and Sepsis-2 criteria and compared it with those of CRP and PCT. When comparing the diagnostic performance under Sepsis-3 definition, MDW was not inferior to CRP or PCT in terms of AUC. Regarding the sensitivity with the optimal cutoff from the cohort, MDW showed a higher sensitivity than other markers. For the reason that monocytes are rapid responders of infection, Crouser ED et al. have suggested MDW as a novel biomarker of sepsis in the ED under the definition of the Sepsis-2 criteria [9]. Another study [10] has shown that MDW was also effective under Sepsis-3 definition.

Studies on MDW suggested MDW’s role as a biomarker in specific conditions [25, 26]. A study on MDW in healthy blood donors [26] has suggested reference interval of approximately 16 to approximately 23, which was different from the manufacturer’s recommendations [14]. Agnello L et al. [25] have tested MDW in the ICU, where patients have more serious medical conditions. They did not suggest the optimal cutoff of MDW in the ICU due to study design limitations (pilot study and small sample size). The median value of MDW was significantly higher in the sepsis group on ICU admission (30.9; IQR, 25.6–36.0) than that in the immune-compromised group (24.9; IQR, 21.4–27.8) in this study. In addition, Agnello L et al. [27] have tested MDW in the ED using Sepsis-2 definition and suggested the cutoff for sepsis diagnosis as 23.5; however, only 4% (n = 88) of the overall population (n = 2215) had sepsis. These suggested that MDW was highly affected by underlying medical conditions and characteristics of study populations.

In this analysis, the AUC of all biomarkers showed fair accuracy in sepsis diagnosis, which are disappointing results compared with those of other studies [11, 28]; this might be because we enrolled patients regardless of their immune status. We included immune-compromised patients (i.e., those with malignancy, those who had received cytotoxic chemotherapy, those with neutropenia, and those with other diseases and drug status affecting the immune status) to reflect real-world practice. Additional analysis was performed according to the patients’ immune statuses due to the high proportion of immune-compromised patients. As we expected, the diagnostic performance of MDW was affected by immune status. From the diagnostic performance of the Sepsis-3 criteria, according to immune status, we observed a trend that the AUC values of the biomarkers studied in immune-compromised patients were lower than those in immune-competent patients. The optimal cutoff value of MDW for predicting sepsis was higher for immune-compromised patients (22.0) than that for immune-competent patients (19.0). The AUC of ROC of MDW was not significantly different from those of other biomarkers in immune-competent patients. However, significant changes in the biomarkers were observed across different septic conditions regardless of the immune status, except for WBC count (S2 Table and Fig 2), suggesting that MDW is affected by septic conditions while being affected by the immune status. The number of immune-compromised patients was relatively higher (more than 50% of the overall population) in our cohort than that in previous studies, where patients with compromised immune cell function accounted for only approximately 17% of the study population [10]. This difference might explain the lower AUC values observed in this analysis compared than those observed in previous reports [9, 10]. The additional analysis of immune-competent patients showed that the AUC (0.73; 95% CI, 0.66–0.80) was comparable to that of previous data (AUC, 0.73; 95% CI, 0.69–0.76) [10]. The AUC of MDW in immune-competent patients was higher than that in immune-compromised patients, suggesting that MDW would be more beneficial in patients without the following conditions: malignancy, chemotherapy, immunosuppression, and organ transplantation. Altogether, MDW may be a useful marker of sepsis but should be interpreted with caution according to the patient’s immune status.

Although, in previous studies, the potential benefit of combining MDW with WBC count [9, 10] was reported, the combined effects of MDW, WBC count, CRP, and PCT were disappointing in this analysis (S3 Table). Even the well-known combination of the sepsis markers CRP and PCT [29] did not improve the AUC (0.74; 95% CI, 0.70–0.79) to beyond that achieved by a single marker (CRP [AUC, 0.75; 95% CI, 0.71–0.78] or PCT [AUC, 0.76; 95% CI, 0.82–0.79]). This suggests that with WBC count and MDW, which can be obtained simultaneously, additional tests for CRP and PCT do not have advantages in sepsis diagnosis. It is important to mention that despite the slightly higher AUC, PCT, and CRP showed a relatively lower sensitivity using the optimal cutoff values of 0.05 (sensitivity of 76.6% [95% CI, 76.4%–76.8%]) and 4.0 (sensitivity of 69.7% [95% CI, 69.5–69.9]), respectively.

A recent study has shown the usefulness of MDW plus clinical scores [30]. Crouser et al. [30] have shown that normal MDW combined with SIRS or qSOFA reduced the probability of sepsis and suggested MDW as complimentary marker to clinical scores. In this analysis, when combined with a clinical score (qSOFA), MDW showed a significantly improved diagnostic accuracy in terms of AUC in the overall population (Table 5). In current guidelines, the suggested algorithm for identifying sepsis uses the qSOFA as the first-line assessment tool. MDW takes a shorter laboratory testing time than CRP or PCT and uses the same blood specimen and test equipment as those in CBC. Moreover, the qSOFA can be calculated in real time in clinical practice. The combination of MDW and qSOFA may provide effectiveness and convenience as an initial screening test.

In addition, we conducted the analysis of biomarkers’ performance using the Sepsis-2 criteria. Among the tested biomarkers, CRP showed the highest AUC of ROC (0.79; 95% CI, 0.75–0.82). When MDW was combined with WBC count, the performance was comparable to that of CRP and PCT. The overall AUC of ROC was not significantly different from that of Sepsis-3 definition.

### Strengths

In previous studies, in which MDW was evaluated, the primary focus was to check the effectiveness of MDW in detecting sepsis and its validation [9, 10] and to compare it with other inflammatory markers, that is, PCT and WBC count [9–11]. We collected not only the CBC data (WBC count and MDW) but also the data of conventional biomarkers, such as CRP and PCT, whereas Polilli et al. [11] have compared MDW with PCT but in a limited number of patients; CRP was not present in that study. Moreover, we compared the biomarkers with qSOFA and the combinations of the biomarkers. We collected data from patients with various immune statuses and performed additional analysis according to the immune status to differentiate patients who would potentially benefit from determining MDW from those who would not.

### Limitations

This study has some limitations that need to be considered while interpreting the results. First, we enrolled patients when the physicians recommended CBC with differential counts; thus, there was a risk of selection bias. In the ED, patients who got laboratory test performed were selected according to the physicians’ decision; the non-infection group in this population was more likely to have an underlying disease (e.g., 34.5% of the patients could have had some kind of malignancy) compared with real controls (healthy). Second, this was a single-center study; hence, there could be high pretest probability of sepsis. The center where the study was conducted is one of the largest cancer centers in South Korea. The sepsis group accounted for 34.2% of the overall population. The median Charlson Comorbidity Index of the overall population was 4 (IQR, 2–6), meaning that they had at least chronic diseases even considering their age. Sepsis occurs more in patients with underlying diseases [31]. Thus, some study results (sensitivity, specificity, etc.) could not be directly applied to general ED populations. However, the primary outcome of this study was to compare the diagnostic performance of biomarkers based on AUC, which does not account for the prevalence of disease. Third, because of the limited information on baseline medical conditions before the ED visit, we used the worst SOFA score in the ED to diagnose sepsis; this might have resulted in incorrect SOFA scores (e.g., patients with previous hepatic, renal, or hematologic diseases could have had a higher SOFA score), which could have resulted in a misclassification bias. In addition, information about baseline organ function is limited for most patients in the ED; hence, the results of this study may reflect real-world practice. Fourth, a large number of subjects were excluded from this analysis because of invalid laboratory results. As we mentioned earlier, this cohort comprised a high proportion of immune-compromised patients, as did the excluded patients. This condition may affect the WBC counts and patient characteristics and make the test results unreliable. Among those with invalid laboratory results (n = 117), immune-compromised patients accounted for 77.8% (n = 91) of the overall population. The number of patients with invalid MDW results was 39 (5.9% of patients with blood sample [n = 666]).

Sepsis is not a single disease, but a clinical syndrome. Hence, no perfect standard exists for sepsis diagnosis, and there is always a risk of misdiagnosis. Considering the risk of overdiagnosis [32], the biomarker of sepsis must be sensitive because of poor clinical outcomes of delayed diagnosis. As earlier intervention (e.g., antibiotics and fluid resuscitation) results in better outcomes in sepsis management, rapid, and easily available screening tools are highly beneficial for patients with sepsis. MDW showed a relatively higher sensitivity, negative predictive value, and better diagnostic performance than conventional inflammatory markers. Hence, when combined with WBC count and qSOFA, MDW could more accurately predict sepsis. With CBC, MDW may help physicians be more observant of sepsis and proceed with further steps required for evaluating the septic condition, thus facilitating early intervention for sepsis.

## Conclusions

MDW, which can be measured along with CBC, has a diagnostic performance comparable to those of conventional biomarkers, such as CRP and PCT; hence, it could be an early marker of sepsis and help decide further evaluation of sepsis. When combined with a clinical score (i.e., qSOFA), a more powerful diagnostic performance could be achieved.

## Supporting information

S1 Table Baseline characteristics of immune-compromised and immune-competent patients. (DOCX)

S2 Table Comparison of biomarkers levels according to immune status by Sepsis-3 definition. (DOCX)

S3 Table Comparison of discriminative performance of MDW combined with other biomarkers according to Sepsis-3 definition. (DOCX)

S4 Table Baseline clinical characteristics of patients visiting the emergency department according to Sepsis-2 definition. (DOCX)

S5 Table Comparison of discriminative performance of MDW combined with other biomarkers for sepsis, according to Sepsis-2. (DOCX)

## Abbreviations

AUC: area under the curve
CBC: complete blood count
CI: confidence interval
CRP: C-reactive protein
ED: emergency department
G-CSF: granulocyte colony-stimulating factor
ICU: intensive care unit
IQR: interquartile range
LR: likelihood ratio
MDW: monocyte distribution width
NPV: negative predictive value
PCT: procalcitonin
PPV: positive predictive value
qSOFA: quick Sequential Organ Failure Assessment
ROC: receiver operating characteristic
SD: standard deviation
SOFA: Sequential Organ Failure Assessment
