# Supplementary material for: Monocyte distribution width compared with C-reactive protein and procalcitonin for early sepsis detection in the emergency department
Source: PLoS One. 2021 Apr 15;16(4):e0250101. doi: 10.1371/journal.pone.0250101 (PMC8049232; doi:10.1371/journal.pone.0250101)
Supplement: S1 Table — (DOCX) [file pone.0250101.s001.docx]

**S1 Table. Baseline characteristics of immune-compromised and immune-competent patients.**

|  | Total | Non-infection | Infection | Sepsis | *P* value |
| --- | --- | --- | --- | --- | --- |
| Immune-compromised^a^ | *n* = 307 | *n* = 95 | *n* = 81 | *n* = 131 |  |
| Age, years, mean (SD) | 61.2(10.9) | 60.0(11.5) | 59.1(11.0) | 63.3(10.9) | 0.012 |
| Sex, male, *n* (%) | 166 (54.1) | 52(54.7) | 39 (48.1) | 75(57.3) | 0.429 |
| CMI, median (IQR) | 5.0 (4-8) | 5.0 (3-7) | 5.0 (4-8) | 6.0 (4-8) | 0.116 |
| SOFA score, median (IQR) | 2.0 (1-3) | 1 (1-2) | 1 (1-1) | 3 (2-4) | <0.001 |
| qSOFA, median (IQR) | 0.0 (0-1) | 0 (0-0) | 0 (0-0) | 0 (0-1) | <0.001 |
| Lactic acid (mmol/L) , median (IQR) | 1.6 (1.1-2.1) | 1.5 (1.0-1.9) | 1.25 (0.8-1.6) | 1.7 (1.3-2.3) | 0.01 |
| Immune-competent^b^ | *n* = 242 | *n* = 134 | *n* = 51 | *n* = 57 |  |
| Age, years, mean (SD) | 56.7(15.5) | 56.3(15.1) | 59.6(16.0) | 63.8(13.0) | <0.001 |
| Sex, male, *n* (%) | 136 (56.2) | 66 (48.5) | 35 (68.6) | 35(61.4) | 0.04 |
| CMI, median (IQR) | 2.0 (3) | 2 (0-3) | 1 (0-3) | 4 (2-5) | <0.001 |
| SOFA score^c^, median (IQR) | 1.0 (1) | 1 (1-2) | 1 (1-1) | 3 (2-5.5) | <0.001 |
| qSOFA^c^, median (IQR) | 0 (1) | 0 (0-0) | 0 (0-1) | 1 (0-1) | <0.001 |
| Lactic acid (mmol/L), median (IQR) | 1.2 (1) | 1.2(0.9-2.0) | 1.1(0.85-1.25) | 1.4(0.9-1.9) | 0.051 |

SOFA, Sequential Organ Failure Assessment; qSOFA, quick Sequential Organ Failure Assessment; ED, emergency department; G-CSF, granulocyte colony-stimulating factor; IQR, interquartile range; SD, standard deviation; CMI, Charlson Comorbidity Index

^a^Immune-competent was defined as patients not immune-compromised.

^b^Immune-compromised is defined as patients with any malignancy, who were treated with G-CSF, with neutropenia, who underwent organ transplantation, or with acquired immunodeficiency syndrome.

^c^ Scores were the values calculated during ED admission.
