# Supplementary material for: Monocyte distribution width compared with C-reactive protein and procalcitonin for early sepsis detection in the emergency department
Source: PLoS One. 2021 Apr 15;16(4):e0250101. doi: 10.1371/journal.pone.0250101 (PMC8049232; doi:10.1371/journal.pone.0250101)
Supplement: S2 Table — (DOCX) [file pone.0250101.s002.docx]

**S2 Table. Comparison of biomarkers levels according to immune status by Sepsis-3 definition.**

|  | Total | Non-infection | Infection | Sepsis | *P* value |
| --- | --- | --- | --- | --- | --- |
| Immune-compromised^a^ | *n* = 307 | *n* = 95 | *n* = 81 | *n* = 131 |  |
| CRP (mg/dL), median (IQR) | 4.7 (1.4-10.8) | 2.6 (0.6-7.8) | 3.8 (1.2-10.0) | 6.7 (2.9-13.4) | <0.001 |
| PCT (ng/mL), median (IQR) | 0.07(0.05-0.23) | 0.05 (0.05-0.16) | 0.05 (0.05-0.10) | 0.12 (0.05-0.87) | <0.001 |
| WBC (×10^3^/μL), median (IQR) | 7.8(4.6-11.6) | 7.5(4.5-11.0) | 7.6 (4.5-11.4) | 8.4 (4.9-13.2) | 0.533 |
| MDW, median (IQR) | 22.7(19.8-26.7) | 21.2 (18.0-24.9) | 22.2 (19.5-25.7) | 24.9(21.4-27.8) | <0.001 |
| Immune-competent^b^ | *n* = 242 | *n* = 134 | *n* = 51 | *n* = 57 |  |
| CRP (mg/dL), median, (IQR) | 0.91 (0.1-6.4) | 0.16 (0.10-0.72) | 6.12 (1.05-10.55) | 7.65 (2.45-18.61) | <0.001 |
| PCT (ng/mL), median, (IQR) | 0.05(0.05-0.21) | 0.05 (0.05-0.05) | 0.05 (0.05-0.32) | 0.26 (0.085-0.735) | <0.001 |
| WBC (×10^3^/uL), median, (IQR) | 8.6 (6.2-11.7) | 7.0 (5.5-10.15) | 9.9 (7.8-14.4) | 11.4(7.5-14.95) | <0.001 |
| MDW, median, (IQR) | 19.2 (16.7-24.0) | 17.5(16.1-20.8) | 20.9 (18.0-24.0) | 23.2(19.5-27.2) | <0.001 |

MDW, monocyte distribution width; CRP, C-reactive protein; PCT, procalcitonin; IQR, interquartile range.

^a^Immune-competent was defined as patients not immune-compromised.

^b^Immune-compromised is defined as patients with any malignancy, who were treated with G-CSF, with neutropenia, who underwent organ transplantation, or with acquired immunodeficiency syndrome.
