# Supplementary material for: Monocyte distribution width compared with C-reactive protein and procalcitonin for early sepsis detection in the emergency department
Source: PLoS One. 2021 Apr 15;16(4):e0250101. doi: 10.1371/journal.pone.0250101 (PMC8049232; doi:10.1371/journal.pone.0250101)
Supplement: S3 Table — (DOCX) [file pone.0250101.s003.docx]

**S3 Table. Comparison of discriminative performance of MDW combined with other biomarkers according to Sepsis-3 definition.**

|  | MDW | MDW and WBC | MDW and CRP | MDW and PCT |
| --- | --- | --- | --- | --- |
| All patients (*n*=549) | | | | |
| AUC (95% CI) | 0.71 (0.66-0.75) | 0.72 (0.67-0.76) | 0.75 (0.71-0.79) | 0.72 (0.68-0.76) |
| Immune-competent^a^ (*n* =242) |  |  |  |  |
| AUC (95% CI) | 0.73 (0.66-0.80) | 0.76 (0.69-0.83) | 0.80 (0.74-0.86) | 0.74 (0.68-0.81) |
| Immune-compromised^b^ (*n* =307) | | | | |
| AUC (95% CI) | 0.66 (0.60-0.72) | 0.65 (0.59-0.71) | 0.67 (0.61-0.73) | 0.71 (0.65-0.77) |

MDW, monocyte distribution width; WBC, white blood cell; CRP, C-reactive protein; PCT, procalcitonin; CI, confidence interval; qSOFA, quick Sequential Organ Failure Assessment.

^a^Immune-competent was defined as patients not immune-compromised.

^b^Immune-compromised is defined as patients with any malignancy, who were treated with G-CSF, with neutropenia, who underwent organ transplantation, or with acquired immunodeficiency syndrome.
