# Supplementary material for: Monocyte distribution width compared with C-reactive protein and procalcitonin for early sepsis detection in the emergency department
Source: PLoS One. 2021 Apr 15;16(4):e0250101. doi: 10.1371/journal.pone.0250101 (PMC8049232; doi:10.1371/journal.pone.0250101)
Supplement: S4 Table — (DOCX) [file pone.0250101.s004.docx]

**S4 Table. Baseline clinical characteristics of patients visiting the emergency department according to Sepsis-2 definition.**

|  | Total (*n* = 549) | Non-infection  (*n* = 161) | Infection  (*n* = 75) | SIRS  (*n* = 68) | Sepsis  (*n* = 245) | *P* value |
| --- | --- | --- | --- | --- | --- | --- |
| Age, years, mean (SD) | 59.2 (13.3) | 58.1 (14.7) | 58.5 (12.5) | 57.3 (11.7) | 60.6(13.0) | 0.134 |
| Sex, male, *n* (%) | 302 (55.0) | 80(50.9) | 40(53.3) | 36(52.9) | 144(58.8) | 0.442 |
| Previous medical condition, *n* (%) | | | | | | |
| Malignancy | 267 (48.6) | 30(18.6) | 34(45.3) | 49(72.1) | 154 (62.9) |  |
| Neutropenia^a^ | 67 (12.2) | 5(3.1) | 8(10.7) | 15(22.1) | 39(15.9) | <0.001 |
| Use of antibiotics^b^ | 73 (13.3) | 7(4.3) | 6(8.0) | 7(10.3) | 53(21.6) | <0.001 |
| Use of G-CSF | 49 (8.9) | 2(1.2) | 6(8.0) | 10(14.7) | 31(12.7) | <0.001 |
| Immune- compromised |  |  |  |  |  |  |
| AIDS or organ transplant | 6 (1.1) | 2(1.2) | 1(1.3) | 1(1.5) | 2(0.8) | 0.953 |
| Chemotherapy^c^ | 117 (21.3) | 12(7.5) | 14(18.7) | 22(32.4) | 69(28.2) | <0.001 |
| CMI, median (IQR) | 4 (2-6) | 2 (1-4) | 4 (2-6) | 4.5 (3-7) | 5 (3-7) | <0.001 |
| SOFA^d^ score, median (IQR) | 1 (1-3) | 1 (1-2) | 1.0 (1-2) | 2 (1-3) | 2 (1-4) | <0.001 |
| qSOFA^d^, median (IQR) | 0 (0-1) | 0 (0-0) | 0 (0-0) | 0 (0-1) | 0 (0-1) | <0.001 |
| Lactic acid^e^ (mmol/L), median (IQR) | 1.4 (1-1.8) | 1.3 (1-1.8) | 1.3 (0.9-1.5) | 1.6 (0.8-2.4) | 1.5 (1.0-2.0) | 0.23 |

SOFA, Sequential Organ Failure Assessment; qSOFA, quick Sequential Organ Failure Assessment; ED, emergency department; G-CSF, granulocyte colony-stimulating factor; IQR, interquartile range; SD, standard deviation; AIDS, Acquired Immunodeficiency Syndrome;CMI, Charlson Comorbidity Index

^a^Neutropenia was defined as neutrophil counts of less than 1,500/μL [13]. ^b^Use of antibiotics was defined as using antibiotics within 7 days before ED visit. ^c^Chemotherapy was defined as taking any cytotoxic chemotherapy within 2 weeks before emergency department visit. ^d^Scores were the values calculated during ED admission. ^e^Lactic acid was the initial value at the ED.
