# Supplementary material for: Monocyte distribution width compared with C-reactive protein and procalcitonin for early sepsis detection in the emergency department
Source: PLoS One. 2021 Apr 15;16(4):e0250101. doi: 10.1371/journal.pone.0250101 (PMC8049232; doi:10.1371/journal.pone.0250101)
Supplement: S5 Table — (DOCX) [file pone.0250101.s005.docx]

**S5 Table. Comparison of discriminative performance of MDW combined with other biomarkers for sepsis, according to Sepsis-2.**

|  | MDW | WBC | PCT | CRP | MDW and WBC |
| --- | --- | --- | --- | --- | --- |
| All patients (*n* = 549) | | | | | |
| AUC (95% CI) | 0.70 (0.66-0.74) | 0.69 (0.65-0.72) | 0.72 (0.68-0.76) | 0.79 (0.75-0.82) | 0.72 (0.68-0.75) |

MDW, monocyte distribution width; WBC, white blood cell; CRP, C-reactive protein; PCT, procalcitonin; CI, confidence interval.
